# Can radiologic parameters used to detect cervical spinal instability be used in patients with ankylosing spondylitis?

**DOI:** 10.1186/s40001-023-01052-3

**Published:** 2023-02-25

**Authors:** Henrik Teuber, Sascha Halvachizadeh, Melvin Muthirakalayil, Luxu Yin, Harry Eisenkrein, Frank Hildebrand, Philipp Kobbe, Kai Sprengel, Ladislav Mica, Hatem Alkadhi, Hans-Christoph Pape, Roman Pfeifer

**Affiliations:** 1 Department of Trauma Surgery and Harald-Tscherne Laboratory, University Hospital Zurich, University of Zurich, Zurich, Switzerland; 2 Department of Orthopedics Trauma Surgery, University Hospital Aachen, RWTH Aachen University, Aachen, Germany; 3 Department of Radiology, University Hospital Zurich, University of Zurich, Zurich, Switzerland

**Keywords:** Ankylosing spondylitis, Cervical spine, Computed tomography, Radiologic parameters

## Abstract

**Introduction:**

Cervical spinal instability can be difficult to detect in the shock room setting even with the utilization of computed tomography (CT) scans. This may be especially true in patients with cervical degenerative disease, such as ankylosing spondylitis (AS). The purpose of this study was to investigate the influence AS has on various radiologic parameters used to detect traumatic and degenerative instability of the cervical spine, to assess if CT imaging in the shock room is diagnostically appropriate in this patient population.

**Methods:**

A matched, case–control retrospective analysis of patients with AS and controls without AS admitted at two level-1 trauma centers was performed. All patients were admitted via shock room and received a polytrauma CT. Twenty-four CT parameters of atlanto-occipital dislocation/instability, traumatic and degenerative spondylolisthesis, basilar invagination, and prevertebral soft tissue swelling were assessed. Since the study was assessing normal values, study patients were included if they had no injury to the cervical spine. Study patients were matched by age and sex.

**Results:**

A total of 78 patients were included (AS group, *n* = 39; control group, *n* = 39). The evaluated cervical radiologic parameters were largely within normal limits and showed no significant clinical or morphologic differences between the two groups.

**Conclusion:**

In this analysis, CT measurements pertaining to various cervical pathologies were not different between patients with and without AS. Parameters to assess for atlanto-occipital dislocation/instability, spondylolisthesis, or basilar invagination in the trauma setting may reliably be used in patients with AS.

## Introduction

Ankylosing spondylitis (AS) is a progressive arthritis that primarily affects the axial skeleton, which often results in multi-segmental fusion of the spine. The loss of mobility in the vertebral column results in a rigid structure that behaves like a long bone. In association with osteoporosis often accompanying AS in the elderly, these individuals are significantly more susceptible to unstable injuries of the spine. Cervical spinal injuries are common, occurring in 3% of major trauma patients [1]. Cervical trauma in AS patients requires particular attention in the acute shock room setting, as the risk of severe neurological complications due to spinal stenosis is greatly elevated [2, 3].

In the polytraumatized shock room setting, CT is the imaging modality of choice for assessing cervical spinal trauma. With sensitivity of 97–100%, it is very sensitive in detecting cervical spinal fractures. However, with respect to detecting ligamentous instability, sensitivity is much lower and has not been well documented [1]. To assess for cervical spinal instability using CT, indirect parameters primarily looking for subtle subluxations must be used.

In AS, multiple studies have shown progressive pathologic changes to the cervical spine in patients with longer disease duration [2]. In patients with AS for at least 20 years, 19.9% of males and 16.0% of females exhibited cervical-predominant pathology [4]. After 25 years of disease, 75% of AS patients had cervical spine involvement [5]. Additionally, patients with early AS exhibit cervical-predominant pathology in 5.2% of cases. Morphologically, physiologic cervical lordosis may regress and pathologic cervical kyphosis, due to spondyloarthropathy, may develop in AS patients. Patients unload the inflamed facet joints by assuming a kyphotic posture. Ultimately, with increasing syndesmophyte formation and eventual ankylosis, the cervical spine assumes a fixed kyphotic deformity [6, 7].

To assess if potentially subtle cervical traumatic instability in patients with AS can be detected in the shock room setting, it is important to understand if the morphologic changes that can occur in AS affect the measurability of radiologic parameters used in detecting cervical spinal instability. However, little is known about the effect AS has on characteristic radiologic changes found in common traumatic or degenerative soft tissue pathologies of the cervical spine. It is unknown if the normal values of these parameters are the same in patients with and without AS. We thus hypothesized that the ankylosis, spinal degeneration, and deformity commonly found in AS may affect the normal values of important radiologic parameters used in detecting cervical instability including atlanto-occipital dislocation, spondylolisthesis, and basilar invagination. To assess this hypothesis, an analysis of 24 commonly used radiologic parameters were measured in cervical CTs of non-injured patients with AS and non-injured controls without AS.

Finally, it is important to note that this study design was applied in the setting of acute injury diagnostics in a polytraumatized shock room setting. If CT imaging remains unclear, and cervical spinal instability cannot be ruled out, prompt additional MRI or functional conventional imaging to assess for ligamentous injury is required after the patient has been stabilized, as these imaging modalities are much more sensitive in detecting potentially unstable ligamentous injuries of the cervical spine [1].

## Patients and methods

The study was approved by the respective local ethics committees of the two level 1 trauma centers. All patients were treated at two level 1 trauma centers between 2009 and 2019. All patients were admitted via shock room and received a polytrauma CT. Patients may have had multiple injuries, but were included only if they had no injuries to the cervical spine, since this study was designed to assess the applicability of normal limits of various radiologic parameters used in assessing for potential cervical spinal instability. In the study group, patients with a confirmed diagnosis of AS (fulfillment of the modified New York criteria for AS or ASAS criteria for axial SPA) were included. Study group patients were matched 1:1 for age and gender with patients without AS in whom cervical spinal injuries were ruled out.

### Cervical spine CT examinations

**Fig. 1:**
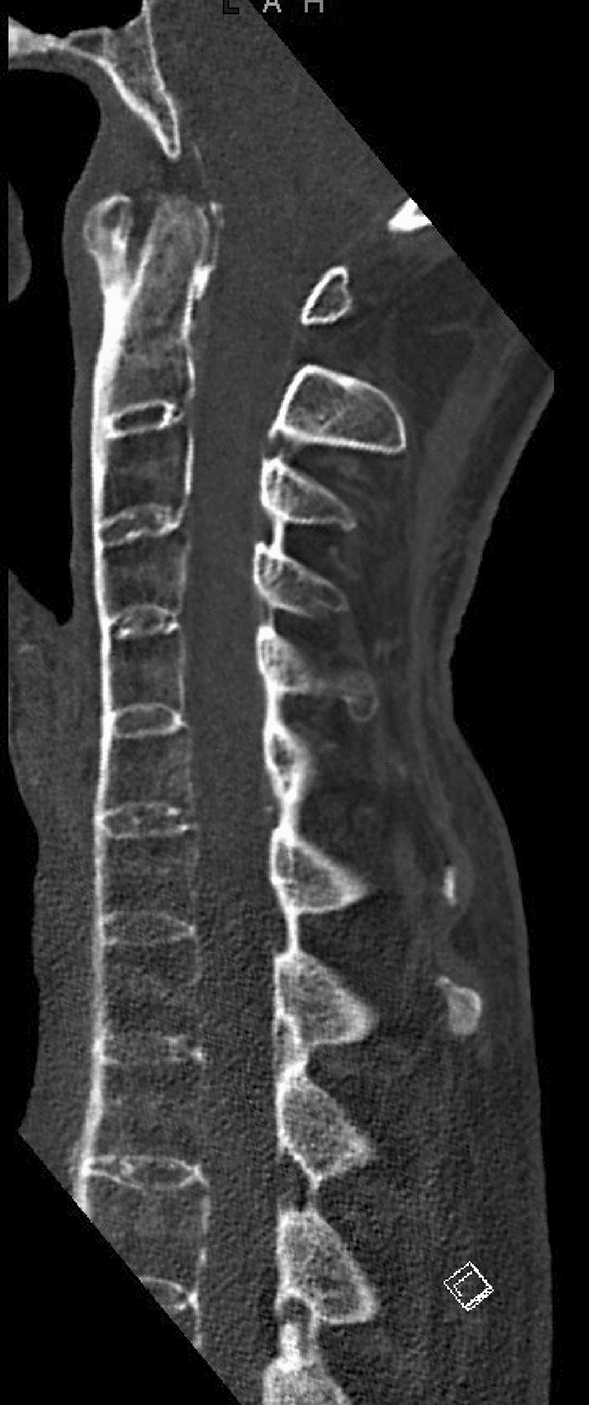
Sagittal plane CT of the cervical spine of a patient in the AS group, an 80-year-old male

All patients underwent scans by multi-detector row CT of the cervical spine. Axial images were reconstructed at a section thickness of 1 mm and an increment of 0.7 mm. Axial, coronal, and sagittal reformations were obtained. Twenty-four radiologic parameters summarized in Table 1 were measured and evaluated by three independent orthopedic surgeons who were blinded to clinical data. An example of a sagittal plane CT of the cervical spine of a patient in the AS group is shown in Fig. 1.

**Table 1:** Brief description and method of measurement of the selected radiologic parameters

BAI	The distance between the basion and the tangent of the posterior border of the axis
BDI	The distance between the basion and the tip of the odontoid process
C2–C3 angle (endplate method)	Angle between lines tangent to the inferior endplate of C2 and C3
C2–C3 angle (posterior method)	Angle between lines tangent to the posterior vertebral body of C2 and C3
Lee X-Line	Lines of basion to the arch of C2 and opisthion to the postero-inferior edge of C2. When both lines do not intersect C2 and C1, respectively, dissociation is suspected
Power ratio 1	The ratio of basis-arc C1/dens-opisthion. A ratio greater than 0.9 is considered pathological and suggestive of atlanto-occipital instability
Power ratio 2	The ratio of basis-arc C1/C1 ventral-opisthion
Chamberlain line	The line connects the posterior end of the hard palate and the posterior lip of the foramen magnum
McCrae’s line	The line of the opening of the foramen magnum
PADI	The posterior interval between atlas and dens
Soft-tissue shadow of C3 and C6	The distance between the anterior aspect of the vertebral body baseplate to the trachea
Endplate tilt of C4–C5 and C5–C6	Angle of lines drawn along the inferior endplate of C4–C5 and C5–C6
Displacement of C4–C5 and C5–C6	The displacement of the postero-inferior edge of the superior vertebral body to the posterosuperior edge of the inferior vertebral body

### Statistics

Data were analyzed using the SPSS software package version 20.0 for Windows (SPSS Inc., Chicago, IL, USA). Descriptive statistics are presented as mean and standard error of the mean (SEM). Statistical distribution of all the data was assessed by both the Shapiro–Wilk and Kolmogorov–Smirnov tests. An independent *t*-test was used to calculate statistical differences between the AS group and the control group. A *p*-value less than 0.05 was considered statistically significant.

## Results

Overall, 78 patients were included in the study. The AS study group consisted of 35 male and 4 female patients with an average age of 66.2 years and was matched on a 1:1 basis with healthy controls. AS patients had a mean New York criteria sacroiliitis grade of 2.89 (SD 0.76). Both groups were demographically very similar. However, steroid use was unsurprisingly significantly higher in the AS vs. control study group (10.3% vs. 0.0% of patients, *p* = 0.044). Study demographics are shown in Table 2. Overall, the radiologic parameters measured were within normal limits and showed little variation between the groups. None of the 24 CT-based parameters measured were statistically or clinically different between the groups and are summarized in Table 3.

**Table 2:** Demographics

	Control	Ankylosing spondylitis	*p*
*n*	39	39	
Age (yrs, mean (SD))	66.2 (14.4)	66.4 (14.2)	Matched
Female (*n*, %)	4 (10.3)	4 (10.3)	Matched
Height (cm, mean (SD))	174.5 (6.0)	172.4 (4.7)	0.169
Weight (kg, mean (SD))	80.7 (11.1)	77.4 (13.8)	0.334
BMI (mean (SD))	26.5 (3.1)	26.2 (4.5)	0.522
Diabetes mellitus (*n*, %)	9 (23.1)	12 (30.8)	0.450
Corticosteroid therapy (*n*, %)	0 (0.0)	4 (10.3)	0.044
Osteoporosis (*n*, %)	2 (5.1)	3 (7.7)	0.649

**Table 3:** Radiologic parameters of various ligamentous injuries

	Control	Ankylosing spondylitis	*p*-value
*n*	39	39	
BDI (mm, mean (SD))	5.3 (1.6)	5.1 (2.0)	0.544
BAI (mm, mean (SD))	5.6 (2.2)	4.9 (2.4)	0.180
Basis–C1 (mm, mean (SD))	32.8 (2.4)	31.7 (3.0)	0.092
Dens–opisthion (mm, mean (SD))	36.4 (3.0)	36.7 (3.1)	0.692
Powers ratio 1 (mean (SD))	0.90 (0.08)	0.87 (0.10)	0.080
C1–opisthion (mm, mean (SD))	42.5 (3.5)	42.8 (3.4)	0.690
Powers ratio 2 (mean (SD))	0.78 (0.07)	0.74 (0.07)	0.059
Basis–C2 (mm)	42.05 (3.32)	40.76 (3.39)	0.092
C2–ophistion (mm)	44.49 (5.96)	43.90 (5.34)	0.644
Lee X-line ratio	0.96 (0.17)	0.94 (0.13)	0.484
Pathological X-line (*n* (%))	11 (28.2)	11 (28.2)	1.000
Chamberlain line length (mean (SD))	82.07 (3.99)	81.58 (3.75)	0.591
Pathological chamberlain line (*n* (%))	7 (17.9)	8 (20.5)	1.000
McCrae’s line length (mean (SD))	37.60 (2.56)	37.37 (3.57)	0.747
Pathological McCrae's line (*n* (%))	0 (0.0)	0 (0.0)	1.000
PADI (mm, mean (SD))	21.3 (2.3)	21.0 (2.1)	0.523
C2–C3 angulation (endplate) (°, mean (SD))	1.9 (5.2)	1.8 (5.6)	0.910
C2–C3 angulation (posterior wall) (°, mean (SD))	6.3 (6.6)	8.0 (7.0)	0.272
End plate tilt C4–C5 (°, mean (SD))	− 1.4 (5.9)	1.6 (8.6)	0.082
End plate tilt C5–C6 (°, mean (SD))	1.8 (5.6)	2.6 (7.4)	0.597
Displacement C4–C5 (mm, mean (SD))	0.6 (1.1)	0.4 (1.2)	0.642
Displacement C5–C6 (mm, mean (SD))	0.3 (0.9)	0.4 (1.0)	0.651
Soft-tissue shadow C3 (mm, mean (SD))	6.1 (4.3)	4.6 (2.6)	0.070
Soft-tissue shadow C6 (mm, mean (SD))	12.4 (4.0)	13.8 (3.5)	0.107

### Atlanto-occipital dislocation/instability parameters

The BDI and BAI were both well within normal limits (< 8.5 mm) and similar in both the AS and control groups (5.1 vs. 5.3 mm, *p* = 0.544 and 4.9 vs. 5.6 mm, *p* = 0.180, respectively). Both methods of measuring Powers Ratio were also similar and within normal limits (< 0.9) in the AS and control groups (0.87 vs 0.90, *p* = 0.080 and 0.74 vs 0.78, *p* = 0.059, respectively). Eleven patients showed pathological Lee X-Line parameters in both study groups (28.2%, *p* = 1.00). Further, we looked at the lengths of the individual lines used to assess Powers Ratio and Lee X-Lines as well as measuring a Lee X-Line ratio to see if we could find any subtle anatomic variation between the two groups. This analysis, however, also showed no differences between the groups. The mean posterior atlanto-dens interval (PADI) was well within normal limits (> 14 mm) and nearly identical in the AS and control groups (21.0 vs. 21.3 mm, *p* = 0.523).

### Basilar invagination parameters

Both Chamberlain’s and McCrae’s lines showed similar results in both the AS group and healthy controls. Seven patients in the control group and 8 patients in the AS group had a pathological Chamberlain line (17.9% vs. 20.5%, respectively, *p* = 1.00). There were no patients with a pathological McCrae’s line in either group (0%, *p* = 1.00). As with the atlanto-occipital parameters above, line lengths were also compared to observe potentially subtle anatomic differences, but no differences between the groups were observed.

### Degenerative/traumatic spondylolisthesis parameters

Endplate tilt at C2/3 (1.8° vs 1.9°, *p* = 0.910), C4/5 (1.6° vs − 1.4°, *p* = 0.082) and C5/6 (2.6° vs 1.8°, *p* = 0.597) were similar and stable (< 11°) between the AS and control groups. However, a consistent, but subtle trend toward more kyphotic angulation, especially at the C4/5 level, was seen in the AS group. Displacement at the level of C3 and C6 were less than 1 mm in both the AS and control patients. Finally, soft tissue swelling at C3 (4.6 vs 6.1 mm, *p* = 0.070) and C6 (13.8 vs 12.4 mm, *p* = 0.107) were within normal limits (C3 < 7 mm, C6 < 21 mm) and similar in both the AS and control groups, respectively.

## Discussion

In this study, despite known morphologic changes to the cervical spine in the setting of AS, none of the cervical radiologic parameters measured in patients with AS were different compared to healthy controls. We could therefore not support the study hypothesis that AS may affect normal values of commonly measured radiologic parameters used in diagnosing atlanto-occipital dislocation, cervical spondylolisthesis and basilar invagination.

Cervical trauma in patients with AS is associated with a high risk of potentially severe neurologic complications [8]. Early awareness and recognition of cervical injuries is, therefore especially important in the setting of AS. However, the assessment of cervical instability or fractures with conventional imaging in patients with AS is challenging. Conventional plain radiographs show low efficiency in diagnosing cervical injuries due to diffuse ossification of cervical spinal ligaments, joints and discs [9]. In a retrospective review, Anwar et al. showed that 60% of cervical fracture dislocations in patients with AS were undetectable in initial radiographs [10]. Sensitivity for detecting cervical injuries is much higher in CT with an improved sensitivity of up to 98% [11–13]. Thus, in the acute trauma setting, the indication for conventional radiologic imaging of the cervical spine is limited and CT of the cervical spine is the standard imaging modality of choice when cervical trauma is suspected [14, 15]. This is especially true in the setting of AS.

### Parameters assessing atlanto-occipital stability are unaltered in AS patients

BAI and BDI are helpful for the diagnosis of atlanto-occipital dissociation injuries. Normal values should be less than 12 mm on plain radiographs. An increase in this distance may indicate instability [16]. Multiple studies declared that the accepted ranges of normal values of BDI and BAI on plain radiographs cannot apply to CT images. Rojas et al. [17] argued that the BAI was difficult to reproduce on CT images; the value was found to be highly variable and a number of subjects had BAIs greater than 12 mm. They found that the distance of BDI was < 8.5 mm in the vast majority of 200 cases and the maximum distance recorded was 9.1 mm. Gonzalez et al. [18] also demonstrated a mean BDI of 4.7 mm and a maximum of 9 mm in CT images from healthy individuals. In line with the literature, the BDI and BAI in the control group of our study were well within normal limits at 5.3 (SD 1.6) and 5.6 (SD 2.2) mm, respectively. Neither BDI nor BAI differed in the AS group compared to the control group and were equally well within normal limits.

Changes to the craniovertebral junction in AS with cervical involvement has been well described and is frequently involved in severe AS [19]. All patients with AS underwent structural changes of articulation and/or ligamentous craniocervical structures [19]. The interval of the atlanto-occipital joint and the atlanto-dental joint were decreased in AS patients, but the BDI and the Power’s ratio were not changed [19]. Our results confirm these findings.

Studies of BAI measurements in AS patients are rare. Robust articular ligaments, such as the cruciform ligament, are important for atlanto-axial joint stability [20]. In the pathological process of AS, these ligaments undergo inflammation such as enthesitis, and laxity or rupture with subsequent atlanto-axial subluxation can occur [21]. Furthermore, odontoid pannus formation can lead to atlanto-axial instability [22]. We suspected that excessive kyphosis might also occur at this level, potentially altering various parameters, especially BAI. However, BAI was found within normal limits and not different to the control group. The only pathological values we found in the present study were seen with the Lee’s X-line parameters with pathological findings in 11 (28.2%) patients in both study arms. The hypothesis of altered atlanto-occipital cervical parameters on account of AS induced instability, specifically pathologic kyphosis, could not be supported in this study.

### Parameters assessing basilar invagination and cervical spondylolisthesis remain stable in AS patients

The endplate and the posterior vertebral body tangent measurement methods for C2–C3 angulation were first described by Levine and Edwards [23]. C2–C3 angulation and translation were usually used to evaluate for traumatic spondylolisthesis including Hangman’s fractures [24, 25]. The physiological cervical spine shows a slight lordotic curvature at the level C2–C3 of about -1.9 ± 5.20° [26]. Cervical lordosis in AS patients is decreased and with increasing severity, even kyphotic deformities may occur [6]. However, it is unknown if kyphotic changes in AS are focussed at specific levels, or if the kyphotic changes are equally distributed throughout the cervical spine and thus difficult to detect at individual levels. In this study, the angulation at C2–C3 as well as C4–C5 and C5–C6 were measured to assess for potential underlying spondylolisthetic changes [27]. Any bony pathologic changes of the endplate may influence the measurement of these angles. This is especially true in AS patients, where the endplate border is altered by the presence of syndesmophytes characteristic of the disease [28, 29]. In this study, no changes in single-level angulation were found between the groups. However, a subtle but statistically insignificant trend toward more kyphosis at all levels measured was seen in the AS versus control group. This was most pronounced at the C4/5 level.

Further, results did not differ regardless if measured using the endplate method or the posterior vertebral body line method [27]. We do, however, feel that the posterior vertebral line method may prove more accurate and useful in evaluating the cervical spine in AS, as it measures the angle of lines drawn perpendicular to the posterior vertebral body aspect of the two endplates. The pathological changes of the vertebral edges therefore have less impact on accurate assessment of possible spondylolisthesis. The results suggest that endplate angulation at single vertebral levels remains largely unchanged, despite overall loss of cervical lordosis in AS patients.

Additionally, no differences or pathological values in vertebral displacement (defined as > 3 mm) measured at C4–C5 and C5–C6 were found between the groups, further suggesting that spondylolisthetic degeneration was not present in the AS study group.

Parameters assessing basilar invagination were also identical between the AS and control group. While approximately 20% of patients in both the AS (*n* = 7) and control (*n* = 8) groups had a pathological Chamberlain line, none of the patients in both the AS and control group had a pathological McCrae line. While of no clinical or diagnostic significance, the line lengths were also measured as with the above parameters used to assess for atlanto-occipital dissociation. Here as well, we saw no differences between the groups, suggesting that subtle morphologic changes in the AS group were not present. While common in other arthopathies, such as rheumatoid arthritis, basilar invagination is rare in AS as pannus formation and degenerative destruction of the craniocervical junction has only been documented in late stage disease [21, 22, 30]. Our results are therefore in line with the literature and previous radiologic studies of AS.

It is important to note that with increasing severity of AS, more osteophytic and syndesmophytic changes occur, which may have influenced the accuracy of some of the measured parameters that rely on precise bony landmarks. The severity of AS was not specifically assessed for in this study and is an important study limitation, especially considering that cervical changes are usually found in late stage disease. Since the majority of the AS patients in this study population were older, however, it is likely that a significant portion of the study population was afflicted with more severe disease. Due to the high patient age, the matched study design was useful in accounting for the potentially confounding effect of general spinal degeneration. Further study limitations include the limited size of the study population, and the fact that the cervical parameters, while carefully measured by three board certified orthopedic surgeons, were not evaluated by fellowship-trained musculoskeletal radiologists, potentially affecting the reliability of measured parameters.

Further research is needed to better rapidly assess for ligamentous injuries of the cervical spine in older patients with AS or other pre-existing degenerative disease processes of the cervical spine in a polytraumatized shock room setting. A study with a larger population would be useful to confirm our results. Perhaps one area of future development could include the identification of laboratory parameters specific for ligamentous injuries of the spine as a more rapid adjunct to further imaging diagnostics, which may not be immediately attainable in the polytraumatized patient setting.

## Conclusion

Our analysis showed that while cervical changes can be significant in AS, commonly assessed traumatic and degenerative radiologic cervical spine parameters were not different in patients with AS compared to matched controls without AS. This study affirms the applicability of these radiologic parameters in the acute trauma setting and suggests that their normal values may be used with confidence in patients with AS. However, further study is necessary to confirm these results and improve diagnostic modalities to quickly and accurately assess for cervical spinal injuries in patients with AS.

## Data Availability

The data generated and analyzed for the current study are not publicly available, but are available from the corresponding author on reasonable request.
